# Zika Virus Infection Produces a Reduction on *Aedes aegypti* Lifespan but No Effects on Mosquito Fecundity and Oviposition Success

**DOI:** 10.3389/fmicb.2018.03011

**Published:** 2018-12-18

**Authors:** Isabella Dias da Silveira, Martha Thieme Petersen, Gabriel Sylvestre, Gabriela Azambuja Garcia, Mariana Rocha David, Márcio Galvão Pavan, Rafael Maciel-de-Freitas

**Affiliations:** ^1^Laboratório de Mosquitos Transmissores de Hematozoários, Instituto Oswaldo Cruz, Fiocruz, Rio de Janeiro, Brazil; ^2^Instituto Nacional de Ciência e Tecnologia em Entomologia Molecular, Universidade Federal do Rio de Janeiro, Rio de Janeiro, Brazil

**Keywords:** *Aedes aegypti*, Zika, vectorial capacity, survival, fecundity, disease transmission

## Abstract

A Zika virus (ZIKV) pandemic started soon after the first autochthonous cases in Latin America. Although *Aedes aegypti* is pointed as the primary vector in Latin America, little is known about the fitness cost due to ZIKV infection. We investigated the effects of ZIKV infection on the life-history traits of *Ae. aegypti* females collected in three districts of Rio de Janeiro, Brazil (Barra, Deodoro, and Porto), equidistant ~25 km each other. *Aedes aegypti* mosquitoes were classified into infected (a single oral challenge with ZIKV) and superinfected (two ZIKV-infected blood meals spaced by 7 days each other). ZIKV infection reduced *Ae. aegypti* survival in two of the three populations tested, and superinfection produced a sharper increase in mortality in one of those populations. We hypothesized higher mortality with the presence of more ZIKV copies in *Ae. aegypti* females from Porto. The number of eggs laid per clutch was statistically similar between vector populations and infected and uninfected mosquitoes. Infection by ZIKV not affected female oviposition success. ZIKV infection impacted *Ae. aegypti* vectorial capacity by reducing its lifespan, although female fecundity remained unaltered. The outcome of these findings to disease transmission intensity still needs further evaluation.

## Introduction

*Aedes aegypti* (Diptera: Culicidae) is known as the primary vector of dengue virus (DENV) and chikungunya virus (CHIKV) and was also found recently to be naturally infected with Zika virus (ZIKV), which reinforces the paramount role of this species in arthropod-borne virus transmission (Ferreira-de-Brito et al., 2016; Patterson et al., 2016). The emergence of an arbovirus followed by an outbreak depends upon several factors such as high density of primary vectors, efficient viral replication, and infectivity in vertebrate and invertebrate hosts and the presence of immunologically naïve hosts (Liu-Helmersson et al., 2014). For instance, recent studies concerning virus infectivity demonstrated that a single amino acid substitution in the NS1 gene improved the efficiency of ZIKV transmission from humans to *Ae. aegypti* and thus increasing its prevalence in mosquitoes (Liu et al., 2017; Rossi et al., 2018).

One of the most critical aspects to arbovirus epidemiology is related to the interaction between the genetic backgrounds of insect vectors and virus strains, which may influence vectorial capacity and transmission rates (Weaver and Reisen, 2010). For instance, Brazilian mosquitoes challenged with a ZIKV strain isolated from a patient in New Caledonia exhibited low vector competence (Chouin-Carneiro et al., 2016) but showed high susceptibility when infected with local ZIKV strains (Dutra et al., 2016; Fernandes et al., 2016). Therefore, it is of utmost relevance to investigate the interaction between mosquito vectors and locally isolated virus strains to provide a more realistic dataset.

The biology and behavior of infected mosquitoes have been little explored, although it is an important aspect of vector-borne diseases. Transmission models and the vectorial capacity formula often overlook that arbovirus infections may alter the behavior and/or pose a fitness cost in life-history traits of mosquitoes and thus affect transmission [cf. Kramer and Ciota (2015) for a comprehensive discussion about this topic]. There is a series of experimental data regarding *Plasmodium* and *Anopheles* mosquitoes that could be interpreted as host behavior manipulation by the parasite to enhance its transmission (Rossignol et al., 1986; Koella et al., 1998; Schwartz and Koella, 2001; Robinson et al., 2018). However, very limited information is available regarding the biology of *Aedes* mosquitoes infected with arboviruses. A few reports have demonstrated that DENV and ZIKV are capable to alter the expression of genes potentially involved in host-seeking behavior, especially odorant-binding protein transcripts (Sim et al., 2012; Etebari et al., 2017). The first two evidences of arbovirus impact on *Ae. aegypti* biting rate came from the 1990s, but produced conflicting results. First, there was no evidence of alteration in the biting behavior of *Ae. aegypti* after intrathoracic inoculation of DENV-2 (Putnam and Scott, 1995). Later, it was observed that dengue-infected mosquitoes required more time to feed on blood than uninfected ones (Platt et al., 1997). More recently, some papers reported a negative effect of DENV-2 on *Ae. aegypti* life-history traits, such as reduced lifespan and fecundity, and also an increase in the time to complete a blood meal (Maciel-de-Freitas et al., 2011, 2013; Sylvestre et al., 2013).

Even though negative effects were revealed for dengue virus, little is known about the potential cost of ZIKV infection in vector biology. The recent ZIKV emergence and its explosive outbreak across the Pacific and Latin America caused thousands of cases in cities such as Rio de Janeiro. Therefore, it sounds reasonable to speculate that, occasionally, susceptible mosquitoes are likely to blood feed more than once in ZIKV-infected hosts during the course of an outbreak. Beyond that, opportunities for arboviral exposure in mosquitoes already infected are considerable, since the viral infection persists throughout the insect life. This phenomenon is known as superinfection (Salas-Benito and De Nova-Ocampo, 2015). Considering adult *Ae. aegypti* females have a lifespan of 15–30 days measured by mark, release, recapture studies (Maciel-De-Freitas et al., 2007; Maciel-de-Freitas et al., 2008; David et al., 2009; Maciel-de-Freitas and Lourenço-de-Oliveira, 2009), the likelihood of ingesting blood from two infected hosts during its lifespan and its effects on mosquito-virus interactions has been receiving little attention. The present study aims to investigate the susceptibility of three field *Ae. aegypti* populations to ZIKV, and the infection effects on its life-history traits after mosquitoes were exposed to a single or two ZIKV-infected blood meals.

## Materials and Methods

### Mosquitoes

Eggs were collected through 80 ovitraps (Fay and Eliason, 1966) placed roughly every 25 m each other in each of three different regions in Rio de Janeiro—Barra (22°58′77″ S, 43°23′41″ W), Deodoro (22°51′01″ S, 43°23′52″ W), and Porto (22°53′43″ S, 43°11′03″ W), distant 15–25 km from each other. Ovitraps were installed over an extensive geographic area to assure we sampled the local *Ae. aegypti* genetic variability. *Ae. aegypti* females are considered a limited flyer with mean distance traveled inferior to 200 m (Maciel-De-Freitas et al., 2007), and thus, it is highly unlike that a single mosquito would cross all field sites analyzed here. A minimum of 500 eggs were collected per site and were hatched in the insectary. Adults were maintained at the insectary under a relative humidity of 80 ± 5% and a temperature of 25 ± 3°C, with *ad libitum* access to a 10% sucrose solution. Experiments were performed with F1 generation mosquitoes. A second collection of eggs was done 5–6 months later using the same protocol, and immature mosquitoes were reared to adults to perform a second experimental infection to evaluate the effect of ZIV on *Ae. aegypti* life-history traits.

### Viral Strain

Females were orally challenged with a ZIKV strain isolated from the urine of a patient in Rio de Janeiro. The ZIKV strain belongs to the Asian genotype Rio-U1 (GenBank accession number KU926309) and showed to have high infectivity to Rio *Ae. aegypti* mosquito populations (Fernandes et al., 2016). Viral titers in supernatants were previously determined by serial dilutions in Vero cells, expressed in plaque-forming unit per milliliters (PFU/ml). All the assays were performed with samples containing 3.55 × 10^6^ PFU/ml. Viral stocks were maintained at −80°C until its use.

### Oral Infection With ZIKV

Two rounds of experimental infection assays were conducted, using the same protocol for mosquito superinfection. Thirty-six hours before infection, 6–7 days old inseminated *Ae. aegypti* females from each of the three populations (Barra, Deodoro, and Porto) were separated in 18 cylindrical plastic cages (70 mosquitoes/cage) for blood feeding. Sugar supply was removed 36-h before mosquitoes were challenged with the infective blood meal to increase female’s avidity. The oral infection procedures were performed through a membrane feeding system (Hemotek, Great Harwood, UK), adapted with a pig-gut covering, which gives access to the defibrinated rabbit blood. The infective blood meals consisted of 1 ml of supernatant of infected cell culture, 2 ml of washed rabbit erythrocytes, and 0.5 mM of ATP as phagostimulant. The same procedure and membrane feeding apparatus were used to feed control mosquitoes, but they received a noninfectious blood meal, with 1 ml of cell culture medium replacing the viral supernatant.

### Oral Superinfection With ZIKV

The explosive emergence of ZIKV across the Pacific and Latin America caused thousands of cases in cities such as Rio de Janeiro. Therefore, it is feasible that mosquitoes may feed more than once on ZIKV-infected hosts during their lifespan. Herein, we simulated *Ae. aegypti* superinfection with ZIKV by offering a second infective blood meal to a fraction of the infected mosquitoes. Seven days after the first oral infection (minimum extrinsic incubation period expected for ZIKV) (Roundy et al., 2017), mosquitoes from Deodoro and Porto populations were challenged with a new infective blood meal, using the same viral strain at the same titration (herein called “superinfection”). The other groups (control and infected) received a blood meal without the virus. *Aedes aegypti* individuals from Barra were not included in this procedure, since there were not sufficient egg stocks.

### Experimental Design

Those females that were visually completely engorged after oral infection assays were individualized in cylindrical plastic tubes (a height of 6.5 cm and a diameter of 3 cm) containing moistened cotton overlaid with filter paper as oviposition substrate on the bottom. Tubes were covered on the top with mosquito netting. Once a week, we offered uninfected anesthetized mice to mosquitoes to all groups as blood source. Filter papers were checked for eggs, and those were counted every third day after a blood meal, when a new filter paper was added. Mosquito survival was checked daily at 09:00 h. Each dead mosquito was removed from the plastic tube, and wing length was measured as the distance from the axillary incision to the apical margin, excluding the fringe (Harbach and Knight, 1980).

Infected mosquitoes were kept in an incubator under an artificial photoperiodic regime of 12 h of light and 12 h of dark, with 10% sucrose solution. Temperature was maintained at 28°C, with a relative humidity of 70–80% throughout the course of experiment.

### Vector Competence of Field Populations

In total, 3–10 mosquitoes from each population were sampled at 7 and 14 days postinfection (dpi) and on 7 days post superinfection (dpsi) to check for infection. For that, bodies and heads of 45 sampled individuals were separated before the RNA extraction procedure to assess ZIKV infection and dissemination, respectively. The 44 selected mosquitoes belonged to all three populations: Barra (*n* = 3), Porto (*n* = 22), and Deodoro (*n* = 20), from infected (*n* = 24) and superinfected (*n* = 20) groups.

### Viral Quantification

Total RNA was extracted from mosquitoes with the QIAamp Viral RNA Mini Kit (Qiagen, Hilden, Germany). Viral RNA detection and quantification were performed individually through RT-qPCR with SuperScript^TM^ III Platinum^TM^ One-Step qRT-PCR Kit (Invitrogen, Carlsbad, CA, USA) in QuantStudio 6 Flex Real-Time PCR System (Applied Biosystems, Foster City, CA, USA). Each reaction was made with 600 nM forward primer (5′-CTTGGAGTGCTTGTGATT-3′, genome position 3451–3468), 600 nM reverse primer (5′-CTCCTCCAGTGTTCATTT-3′, genome position 3637–3620), and 800 nM probe (5′FAM-AGAAGAGAATGACCACAAAGATCA-3′TAMRA, genome position 3494–3517), previously published (Ferreira-de-Brito et al., 2016). Cycling conditions were as follows: 95°C for 2 minutes, followed by 40 amplification cycles of 95°C for 15 s, 58°C for 5 s, and 60°C for 30 s. Virus copy numbers were calculated by interpolation onto an internal standard curve made up of a seven-point dilution series (10^2^–10^8^ copies/ml) of *in vitro* transcribed ZIKV RNA (Bonaldo et al., 2016).

### Statistical Analysis

Mosquitoes from the two experimental infection assays were analyzed as belonging to three treatments: controls, which did not feed on ZIKV infectious blood; infected, which received one single infected blood meal on 6–7 days old; and superinfected, which received an additional infected blood meal on 13–14 days old.

The association among survival and ZIKV infection, population and wing length was analyzed using Cox proportional hazard regression models to obtain hazard ratios and 95% confidence intervals. First, we computed separate univariate Cox regression analyses with population (Barra, Deodoro, and Porto), treatment (control, infected, and superinfected), and wing length as covariates. Only statistically significant variables according to Wald statistic values were included in the multivariate Cox model. Kaplan-Meier (KM) survival curves were created to each mosquito population according to the different treatments (control, infected, or superinfected). We performed log-rank tests to address the global effects of treatment in each mosquito population. If significant, we confronted infected and superinfected against controls. Significance level was adjusted for multiple comparisons with the Bonferroni criteria. Those analyses were performed in the R environment (R Development Core Team, 2011).

Fecundity was analyzed by considering the first four clutches of eggs laid, as only a small number of females laid eggs when they were more than 4 weeks old, precluding adequate numbers for analysis. We considered two aspects of fecundity: 1) oviposition success: the likelihood of laying at least one egg (at a given clutch) with a logistic analysis that included treatment, population, wing length and clutch number (i.e., age); and 2) fecundity: the egg number of the successful mosquitoes with a repeated analysis. We included clutch number as the repeat and estimated the effects of treatment, wing length and population. These analyses were carried out with the statistical software JMP v. 13.0 (SAS Institute Inc., 2007).

The abundance of RNA ZIKV was not normally distributed (Shapiro-Wilk *W* = 0.4560, *p* < 0.001) and, therefore, the three treatments were compared through Wilcoxon-Mann-Whitney tests in the R environment (R Development Core Team, 2011). Significance level was adjusted for multiple comparisons with the false discovery rate (FDR) method (Benjamini and Yekutieli, 2001). The effect size of comparisons (*r*) was performed with Cohen’s *d* calculations (Cohen, 1988).

## Results

### Oral Infection and Superinfection

A total of 767 *Ae. aegypti* F1 females from three districts of Rio de Janeiro (Barra, Deodoro, and Porto), Brazil, were used in the experiments. From that, 44 individuals were used for vector competence tests, 399 females were orally infected with a local ZIKV strain (345 were infected once and 54 were superinfected, i.e., received a second infective blood meal at 7 days postinfection (dpi) with the same ZIKV strain). Of the 723 mosquitoes monitored daily during fitness cost assays, 90 belonged to Barra (60 infected and 30 controls), 308 to Deodoro (140 infected, 147 controls, and 21 superinfected), and 325 (145 infected, 147 controls, and 33 superinfected) to Porto field populations.

### Vector Competence Tests

**Figure 1:**
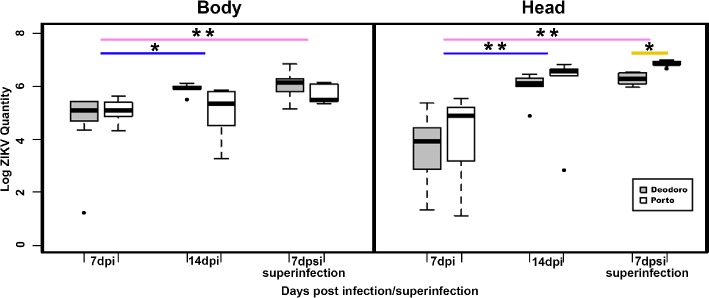
The viral load in the body and head of *Aedes aegypti* mosquitoes infected and superinfected with ZIKV from Deodoro and Porto field populations. Dpi: days postinfection; dpsi: days post superinfection. *p < 0.05; ** *p* < 0.001. Due to low-sample sizes, Barra population data were not included in the analysis.

A total of 45 mosquitoes confirmed the susceptibility of the three populations to the locally isolated ZIKV strain Rio-U1. From that, 44 (97.7%) mosquitoes presented ZIKV at their bodies, and 41 (91.1%) had positive heads. As expected, the number of viral copies increased in bodies and heads over time for Porto and Deodoro. Regarding ZIKV quantification in bodies, although marginal and not significant, we observed a difference between Deodoro and Porto infected mosquitoes occurred at 14 dpi, with the former population presenting more ZIKV genome copies (*W* = 23, *p* = 0.032; Figure 1).

However, no significant difference in the number of viral copies was detected when the heads of Porto- and Deodoro-infected mosquitoes were compared at 7 and 14 dpi (Figure 1). Superinfected mosquitoes from Deodoro and Porto had statistically similar viral copies in their bodies. Interestingly, superinfected *Ae. aegypti* from Porto showed significantly more ZIKV RNA loads at their heads at 7 days post superinfection (dpsi) than Deodoro population, reaching 10^7^ copies (*W* = 0, *p* = 0.007; Figure 1).

### Survival

Wing length had no detectable influence in mosquito survival (*W* = 0.15, df = 1, *p* = 0.7), while population and treatment exhibited significant effects (*W* = 28.3, df = 2, *p* < 0.01; *W* = 13.8, df = 2, *p* < 0.01, respectively) and thus were included in the multivariate analysis. The multivariate Cox model confirmed that populations exhibited different survival curves under laboratory conditions, despite the ZIKV infection (Table 1). In total, only eight individuals survived longer than 60 dpi, five from Deodoro and three from Porto. The median survival was 16 (95% confidence interval, CI: 15–19), 23 (95% CI: 22–25), and 20 (95% CI: 19–23) days to the Barra, Deodoro, and Porto populations, respectively. The Cox model also revealed that the treatment (i.e., exposure to ZIKV) produced a decrease in survival, regardless of population. These results suggest that ZIKV infection negatively affects the longevity of *Ae. aegypti*, with a stronger effect (i.e., a higher hazard ratio) in superinfected mosquitoes (Table 1).

**Table 1:** Associations between mosquito survival and population (Barra, Deodoro, and Porto) and also survival and treatment (control, infected, and superinfected).

Variable	Regression coefficient	Hazard ratio (95% CI)	*z*	*p-value*
**Population**				
Barra	–	1.00 (reference)	–	–
Deodoro	−0.62	0.54	−4.94	<0.01
Porto	−0.42	0.65	−3.44	<0.01
**Treatment**				
Control	–	1.00 (reference)	–	–
Infected	0.20	1.22	2.51	0.01
Superinfected	0.42	1.53	2.85	0.004

**Figure 2:**
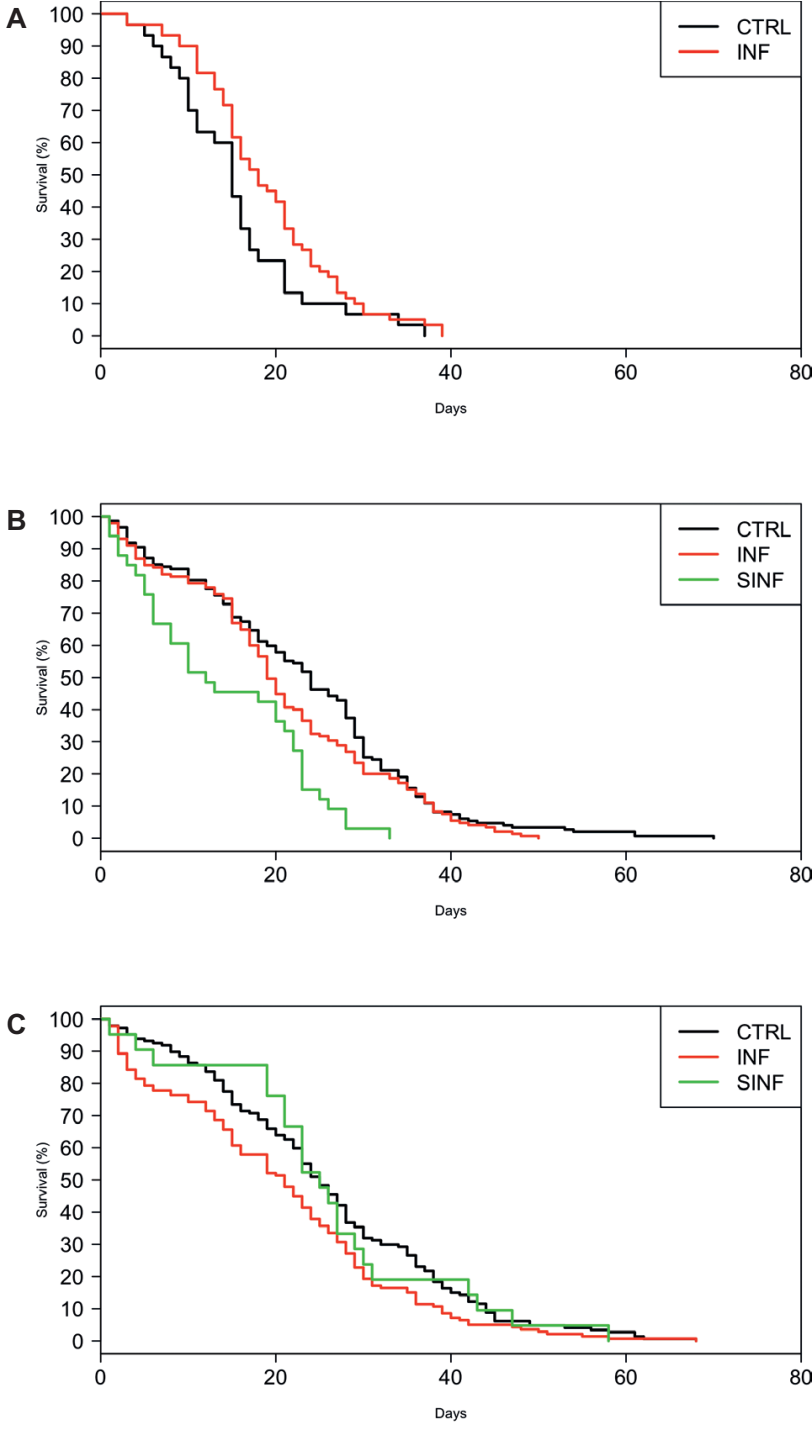
*Aedes aegypti* survival curves according to treatment (control, infected, and superinfected). Data for **(A)** Barra, **(B)** Porto, and **(C)** Deodoro populations. CTR: control (noninfected mosquitoes); INF: infected mosquitoes; SINF: superinfected mosquitoes.

The ZIKV infection effect on mosquito survival was further investigated with survival curves (Figures 2A–C) and paired comparisons between infected and control groups. The treatment did not alter the survival of Barra mosquitoes ( *χ* ^2^ = 3.5, df = 1, *p* = 0.062), but produced significant effects on *Ae. aegypti* populations from Deodoro ( *χ* ^2^ = 8, df = 2, *p* = 0.01) and Porto ( *χ* ^2^ = 20.3, df = 2, *p* < 0.01). In Deodoro, ZIKV infection (but not superinfection) altered mosquito mortality ( *χ* ^2^ = 7.6, df = 1, *p* = 0.01). However, a different trend was observed for Porto mosquitoes—only the superinfection promoted a reduction in survivorship when compared to the control group ( *χ* ^2^ = 22.1, df = 1, *p* < 0.001).

### Oviposition Success

The likelihood of *Ae. aegypti* females laying at least one egg per gonotrophic cycle was strongly affected by mosquito age, with older insects having lower oviposition success (Table 2). Deodoro females exhibited a significant higher oviposition success (an average of 50% considering the four first batches), while the mosquitoes from Barra and Porto exhibited lower averages (27% for both populations). On the other hand, the treatment (control, infection, or superinfection with ZIKV) not affected the oviposition success of *Ae. aegypti* (Table 2).

**Table 2:** Logistic regression analysis of the mosquito population, treatment, wing size, and age when they lay eggs on the success of oviposition of *Aedes aegypti* females.

Variable(s)	df	*χ* ^2^	*p-value*
Age	9	30.52	0.0001
Wing size	3	3.56	0.312
Population	6	44.01	0.0001
Treatment	6	1.47	0.961
Population × treatment	9	0.98	0.999
Population × age	18	41.26	0.004
Population × wing size	6	0.83	0.991
Age × treatment	18	14.26	0.711
Wing size × treatment	6	4.21	0.648
Wing size × population × treatment	9	3.96	0.914

### Fecundity

Fecundity analysis considered the egg number of *Ae. aegypti* females that laid at least one egg. Interestingly, the number of eggs laid by females presented a slight increase over time (i.e., with age), from 49.2 in the first clutch to 55.1 in the fourth, in average (Table 3). Mosquito population, treatment and wing size had no effect on the number of eggs laid by *Ae. aegypti* females (Table 3).

**Table 3:** Repeated measure analysis (with clutch taken as the repeat) of the number of eggs laid by *Aedes aegypti* females.

Variable	Num df	Den df	*F*	*p-value*
Population	6	48	1.648	0.154
Treatment	6	48	1.503	0.197
Wing size	3	24	2.63	0.073

## Discussion

The vectorial capacity (VC) is defined as the entomological component of the basic reproduction rate (*R*_0_) of vector-borne diseases such as malaria, dengue, and Zika viruses (Service, 1993). Therefore, appropriate estimates of VC parameters may provide valuable insights into disease epidemiology and also yield the establishment of more efficient vector control activities to mitigate transmission (Brady et al., 2016). Previous studies have shown that a given pathogen can modify life-history traits of vectors and, therefore, directly influence the VC and ultimately the R_0_ (Scott and Lorenz, 1998; Moncayo et al., 2000; Martin et al., 2010; Vezilier et al., 2012). This article describes the effects of a locally isolated ZIKV strain (Asian genotype Rio-U1) on the biology of three *Ae. aegypti* field populations from Rio de Janeiro. Our results showed that a single (infection) or two ZIKV infected blood meals (superinfection) posed significant effects on the longevity, but not on the fecundity of female mosquitoes. Moreover, mosquito populations differed in their response to virus infection regarding survival rates and viral loads in the body and head.

Shortened lifespan due to pathogen infection was observed in different insect models, such as *Culiseta melanura* orally challenged with Eastern equine encephalomyelitis virus, *Aedes albopictus* infected with chikungunya, and *Anopheles stephensi* mosquitoes infected with *Plasmodium berghei* (Scott and Lorenz, 1998; Dawes et al., 2009; Martin et al., 2010). Negative effects on *Ae. aegypti* longevity were also detected after challenging females with a DENV-2 strain that has never circulated in the region where mosquitoes were collected (Maciel-de-Freitas et al., 2011; Sylvestre et al., 2013). Interestingly, our study pointed that one of the three tested populations (Barra) not had any reduction in lifespan due to ZIKV infection, while the other two populations exhibited an increase in mortality when infected (Deodoro) or superinfected (Porto). However, considering that Barra had only a few mosquitoes, conclusions about this population must be taken carefully. Although we did not accessed all the variables that affect the VC, mortality rate is one of the most important entomological parameters for its estimation (Luz et al., 2003) and thus is reasonable to assume that changes on mosquito lifespan may affect disease transmission under natural settings.

Despite distant each other less than 25 km, the response of mosquito populations to the laboratory environment and to ZIKV infection differed substantially. Deodoro mosquitoes had a higher significant lifespan than Porto and Barra populations, while Porto had a greater survival than Barra, despite the infection status. The treatment also produced different outcomes in Deodoro and Porto populations. The mortality of infected Deodoro mosquitoes decreases in comparison with the control group, while only the superinfected females from Porto had a significant decline in survival. We hypothesized that the higher mortality in superinfected *Ae. aegypti* from Porto is related to a greater viral load in the heads when contrasted to Deodoro mosquitoes. Virulence is tightly coupled to parasite load in *An. stephensi* and to higher RNA copy numbers in Drosophilidae, which means that the extent of the harm to a host might be partially explained by pathogen accumulation (Dawes et al., 2009; Longdon et al., 2015).

Vector competence seems to be influenced by specific interactions between mosquitoes and arbovirus genotypes. By challenging three isofemale families of field-derived *Ae. aegypti* from Thailand with three contemporaneous low-passage DENV-1 isolates, Lambrechts et al. (2009) evinced vector competence is likely governed to a large extent by virus and mosquito genetic interactions in natural populations. Moreover, the susceptibility of *Ae. aegypti* to pathogens is a highly dynamic feature, dependent on both genetic and environmental factors. Thus, it is expected that both vector competence and fitness cost of an infection also vary among geographically close field mosquito populations (Gubler et al., 1979; Failloux et al., 2002; Tabachnick, 2013; Severson and Behura, 2016). Nuclear markers of *Aedes aegypti* populations collected in Rio de Janeiro, including areas overlapping Deodoro, Barra, and Porto, revealed multiple introductions and extensive gene flow among populations. However, a strong spatial structuring was found considering mitochondrial markers and 25 genes related to mosquito immune response and insecticide resistance (Rašić et al., 2015). Therefore, these data may explain why the populations studied here exhibited different outcomes in longevity and superinfected with ZIKV.

Regardless of the extensive studies about the immune pathways activated in mosquitoes during a single flavivirus exposure, it remains unclear whether the immune system activation through a primary virus infection has any effect on a secondary infection (Xi et al., 2008; Souza-Neto et al., 2009; Sim and Dimopoulos, 2010). In the case of the infection with phylogenetic closely related viruses (at least from the same genus) in a single specimen, a superinfection exclusion is expected (i.e., a primary virus infection inhibits a secondary infection), since common host immune factors might be formerly activated by the first infection (Bolling et al., 2012). Mosquito cells previously infected with DENV and then exposed to the four DENV serotypes displayed a reduction in the viral titer (Igarashi, 1979; Kuno, 1982). A similar outcome was observed in *Culex quinquefasciatus* mosquitoes with a persistent infection by a *Culex* flavivirus and subsequently exposed to West Nile virus (WNV) (Bolling et al., 2012). However, Kuwata et al. (2015) found no evidence of superinfection exclusion of Japanese Encephalitis virus or DENV in cells previously infected with a *Culex* flavivirus. In the present study, no evidence of superinfection exclusion was identified, since viral titers did not decrease at 7 dpsi. Indeed, mosquitoes from Porto had a sharp decline on their survival when superinfected.

One limitation of this study is the impossibility of analyzing the effect of arbovirus genotypes in the susceptibility and life-history traits of the mosquitoes, since we infected them with a single ZIKV local strain. Genomic analyses of ZIKV epidemic strains of 2016 revealed the coexistence of at least seven phylogenetically diversified virus clusters circulating in Brazil (Shi et al., 2016; Wang et al., 2017). Although there is no information available about the diversity of ZIKV in small geographic scales such as those studied here, high levels of genetic variability of the flavivirus WNV among local strains were observed in near counties of the New York State infecting *Culex* mosquitoes (Ehrbar et al., 2017). In line with the idea of vector competence being population-specific, the possible genetic variability of ZIKV strains could contribute to the prevalence and transmission on local levels.

Several papers reported the impact of pathogen infection on host fecundity, which associated with a reduction in survival rate, would negatively impact VC by reducing the offspring size (Kramer and Ciota, 2015). For instance, *An. stephensi* and *Anopheles gambiae* seem to produce smaller egg batches after infected with *Plasmodium yoelii nigeriensis* (Hogg and Hurd, 1995; Jahan and Hurd, 1998). WNV-infected *Culex tarsalis* presented smaller egg rafts, mainly in the first oviposition cycle (Styer et al., 2007). *Aedes aegypti* females exposed to DENV-2 exhibited an impairment in fecundity, which varied over mosquito lifespan. Overall, DENV-2 infection seems to interfere with mosquitoes’ fecundity by both reducing egg-laying success and batch sizes (Maciel-de-Freitas et al., 2011; Sylvestre et al., 2013). Bearing in mind that ZIKV reaches mosquito ovaries on the second day postinfection (Li et al., 2017), we sought that the virus would exert a resembling impact over fecundity traits on the first clutch onward. Surprisingly, ZIKV infection did not influence directly on the oviposition success and fecundity, although increased mortality early after infection may result in lower reproduction rates.

*Aedes aegypti* vector competence seems to be genetically determined, which means that coadaptation between mosquitoes and viruses in a local setting may provide a more efficient transmission by sympatric vector genotypes with lower fitness cost (Lambrechts et al., 2009). Overall, our findings suggest that ZIKV infection causes a reduction in *Ae. aegypti* survival but did not alter fecundity, different from what has been observed for DENV and other arboviruses. It is worth mentioning, however, that mosquito populations varied in their response to ZIKV infection considering survival and viral loads, highlighting that fitness outcomes may be governed by the interaction between host and parasite genotypes. For example, superinfected mosquitoes from one of the populations exhibited more ZIKV copies in the head at 7 dpsi and increased mortality. These different outcomes toward field-derived *Ae. aegypti* might provide additional information regarding local epidemiological settings. In conclusion, ZIKV infection yields a reduction in *Ae. aegypti* survival but produced no effects on mosquito fecundity and oviposition success. Therefore, the presence of ZIKV negatively affected the *Ae. aegypti* vectorial capacity by reducing mosquito lifespan.

## Ethics Statement

ZIKV-infected and uninfected mosquitoes were blood fed once a week on anesthetized mice. This study was carried out in accordance with the recommendations of Fiocruz Ethical Committee for Animal Use. The protocol was approved by the Fiocruz Ethical Committee for Animal Use (CEUA LW-32/14).

## Author Contributions

IS, GG, MGP, and RM were responsible for the conception and design of study; IS, MTP, GS, and MTP were responsible for the acquisition of data; MD, MGP, and RM performed the data analysis; IS, MD, MGP, and RM wrote the manuscript. All authors participated in interpretation of the findings, and all authors read and approved the final version of the manuscript. All authors confirm that the content has not been published elsewhere and does not overlap with or duplicate their published work.

### Conflict of Interest Statement

The authors declare that the research was conducted in the absence of any commercial or financial relationships that could be construed as a potential conflict of interest.
